# Complete Genome Sequences of Two *Rhodococcus* sp. Strains with Large and Linear Chromosomes, Isolated from Apple Rhizosphere

**DOI:** 10.1128/MRA.00159-21

**Published:** 2021-06-17

**Authors:** Sarah Benning, Nicolò Brugnone, Roberto Siani, Susanne Kublik, Michael Schloter, Viviane Rad

**Affiliations:** a Research Unit for Comparative Microbiome Analysis, Helmholtz Center Munich, German Research Center for Environmental Health, Neuherberg, Germany; b Chair for Soil Science, Technical University of Munich, Freising, Germany; University of Maryland School of Medicine

## Abstract

Members of the genus *Rhodococcus* are usually able to catalyze a number of processes, which are of great interest for ecosystem performance as well as biotechnology. Here, we report the complete genome sequences of two *Rhodococcus* strains that were isolated from rhizosphere soil from an apple orchard in northern Germany.

## ANNOUNCEMENT

The genus *Rhodococcus* is known for its immense metabolic diversity, such as the production of a large variety of secondary metabolites, including antibiotics, but also for its capacity to catalyze the degradation of aromatic compounds, for example, pesticides (1, 2). Although *Rhodococcus* is known for its large genome size, with possible linear chromosomes and megaplasmids (3), many of the genes coding for enzymes that catalyze the catabolism and metabolism of organic molecules are located on several large or small, mainly linear plasmids (4).

Here, we report the complete genome sequences of *Rhodococcus* sp. strain R79 and Rhodococcus koreensis strain R85, which were isolated from the rhizosphere of young M26 apple rootstocks planted on former grass soil in Ellerhoop, in northern Germany (53°42′51.7″N, 9°45′12.5″E), in 2018 (experimental design as in reference 5). Complete genome sequences should give information on the pathways for the degradation of aromatic compounds, as well as the absence of pathogenicity islands. The isolates were obtained by plating serial dilutions of slurries from rhizosphere soil on actinomycete isolation agar (Sigma-Aldrich, Darmstadt, Germany) and were selected for their ability to grow in minimal medium with benzoic acid as the only carbon source. Genomic DNA for sequencing was obtained using a kit-based protocol (Genomic-tip 20/G kit; Qiagen, Hilden, Germany), after cultivating isolates for 2 days at 28°C in actinomycete broth (Sigma-Aldrich). The genomes were sequenced on a Sequel platform (Pacific Biosciences [PacBio], Menlo Park, CA). Genomic DNA was sheared according to the protocol for preparing multiplexed microbial libraries using SMRTbell Express template preparation kit v2.0 with Covaris g-TUBEs and then was concentrated using AMPure PB beads (PacBio). Libraries were prepared using the aforementioned SMRTbell Express template preparation kit v2.0 and the barcoded overhang adapter kit 8A/8B and then were loaded onto a single-molecule real-time (SMRT) cell following the instructions for diffusion loading (PacBio). No size selection was performed here because this would result in possible plasmid loss.

The demultiplexed data from sequencing were assembled using the Microbial Assembly pipeline implemented in SMRT Link v8.0.0.80529 (PacBio), which includes the circularization process. Parameters differing from the default settings were as follows: for R79, seed coverage of 40 and estimated genome size of 8.7 Mb; for R85, seed coverage of 35 and estimated genome size of 8.9 Mb. No rotation of the contigs was performed. The resulting contigs were annotated using the NCBI Prokaryotic Genome Annotation Pipeline (PGAP) (6). Quality and completeness were controlled with CheckM v1.0.18 (7) as implemented in KBase (8). Taxonomy was inferred using the Type (Strain) Genome Server (TGYS) (9). Default parameters were used except where otherwise noted.

The number of raw polymerase reads for the SMRT cell was 388,809 reads. The numbers of polymerase reads and *N*_50_ values from the subread statistics were 44,862 reads and 7,477 bp, respectively, for R79 and 69,894 reads and 6,877 bp, respectively, for strain R85. For both strains, the completeness of the genomes was 99.7%. Strain R79 had an overall genome size of 9,866,560 bp (GC content of 67.28%), with one linear chromosome, four linear plasmids, and one circular plasmid (Table 1). In total, 9,028 genes were detected for the genome of R79. For strain R85, we obtained an even larger genome size of 10,909,875 bp (GC content of 67.18%), with one linear chromosome, one linear megaplasmid, two linear plasmids, and one circular plasmid (Table 1). Here, 9,955 genes in total were detected. From these genes, we conclude that both strains are potentially able to metabolize aromatic compounds and to produce nonribosomal peptides and polyketides. For both strains, we observed more than 6,000 predicted protein features related to secondary metabolism, which again shows the potential of *Rhodococcus* strains for bioremediation purposes. They partly have the potential to support plant growth. Thus, the two *Rhodococcus* strains might be used in the future as biostimulants for plants.

**TABLE 1:** Characteristics of genomes of strains R79 and R85

Strain and contig	Contig length (bp)	GC content (%)	Topology	GenBank accession no.
R79				
Chromosome	8,730,774	67.5	Linear	CP070619
Plasmid 1	468,864	64.9	Linear	CP070618
Plasmid 2	320,146	60.1	Linear	CP070617
Plasmid 3	167,009	64.7	Linear	CP070616
Plasmid 4	102,288	65.0	Circular	CP070615
Plasmid 5	77,479	65.7	Linear	CP070614
R85				
Chromosome	8,983,208	67.7	Linear	CP070609
Megaplasmid	1,241,328	64.8	Linear	CP070610
Plasmid 2	502,657	64.5	Linear	CP070611
Plasmid 3	126,147	65.0	Circular	CP070612
Plasmid 4	56,535	64.0	Linear	CP070613

### Data availability.

The full genome sequences and the raw reads for R79 and R85 have been deposited in the NCBI database under BioProject number PRJNA700828. The genomes can be found in GenBank with the accession numbers CP070614 to CP070619 for R79 and CP070609 to CP070613 for R85. The raw reads have been deposited in the Sequence Read Archive (SRA) with the accession numbers SRX10094454 for R79 and SRX10094455 for R85.
